# Non‑small cell lung cancer carrying PBRM1 mutation suggests an immunologically cold phenotype leading to immunotherapy failure even with high TMB

**DOI:** 10.1038/s41598-022-25050-3

**Published:** 2022-12-01

**Authors:** Xia-ye Miao, Hao Wu, Bi-cheng Ye, Qian-wen Yi, Fang-nan Lin, Yi-lin Wang, Chuan-li Ren, Yan-fang Jiang, Ang Li

**Affiliations:** 1 Department of Laboratory Medicine, Clinical College of Yangzhou University, Yangzhou, Jiangsu China; 2 Department of Oncology, The Affiliated Huai’an Hospital of Xuzhou Medical University and The Second People’s Hospital of Huai’an, Huai’an, Jiangsu China; 3 School of Clinical Medicine, Yangzhou Polytechnic College, Yangzhou, Jiangsu China; 4 Department of Pediatrics, The First Affiliated Hospital of Bengbu Medical College, Bengbu, Anhui China; 5 Key Laboratory of Organ Regeneration and Transplantation of the Ministry of Education, Genetic Diagnosis Center, The First Hospital of Jilin University, Changchun, Jilin China

**Keywords:** Cancer, Computational biology and bioinformatics

## Abstract

High tumor mutation load (TMB-H, or TMB ≥ 10) has been approved by the U.S. FDA as a biomarker for pembrolizumab treatment of solid tumors, including non‑small cell lung cancer (NSCLC). Patients with cancer who have immunotherapy-resistant gene mutations cannot achieve clinical benefits even in TMB-H. In this study, we aimed to identify gene mutations associated with immunotherapy resistance and further informed mechanisms in NSCLC. A combined cohort of 350 immune checkpoint blockade-treated patients from Memorial Sloan Kettering Cancer Center (MSKCC) was used to identify genes whose mutations could negatively influence immunotherapy efficacy. An external NSCLC cohort for which profession-free survival (PFS) data were available was used for independent validation. CIBERSORT algorithms were used to characterize tumor immune infiltrating patterns. Immunogenomic features were analysed in the TCGA NSCLC cohort. We observed that PBRM1 mutations independently and negatively influence immunotherapy efficacy. Survival analysis showed that the overall survival (OS) and PFS of patients with PBRM1 mutations (MT) were significantly shorter than the wild type (WT). Moreover, compared with PBRM1-WT/TMB-H group, OS was worse in the PBRM1-MT/TMB-H group. Notably, in patients with TMB-H/PBRM1-MT, it was equal to that in the low-TMB group. The CIBERSORT algorithm further confirmed that the immune infiltration abundance of CD8+ T cells and activated CD4+ memory T was significantly lower in the MT group. Immunogenomic differences were observed in terms of immune signatures, T-cell receptor repertoire, and immune-related genes between WT and MT groups. Nevertheless, we noticed an inverse relationship, given that MT tumors had a higher TMB than the WT group in MSKCC and TCGA cohort. In conclusion, our study revealed that NSCLC with PBRM1 mutation might be an immunologically cold phenotype and exhibited immunotherapy resistance. NSCLC with PBRM1 mutation might be misclassified as immunoresponsive based on TMB.

## Introduction

Lung cancer is the leading cause of cancer-related death worldwide. Non‑small cell lung cancer (NSCLC) is considered the main pathological pattern of lung cancer, accounting for 85% of all lung cancer cases^1^. Although substantial progress has been made in cancer treatment, the clinical outcomes of NSCLC are still not satisfactory^1,2^. Immune checkpoint blockade (ICB) therapy offers new hope to patients with cancer and has been a pivotal treatment for advanced NSCLC^3^. Nevertheless, some patients have a durable clinical benefit (DCB), whereas others have sustained progression^4,5^. Since then, the opposite outcomes trigger us to look for factors that influence immunotherapy efficacy for NSCLC.

To date, tumor mutational burden (TMB) is the most widely used genetic predictor for immunotherapy^6^. Indeed, the US FDA has approved pembrolizumab treatment of solid tumors based on tumor mutational load, including NSCLC. However, some patients could not achieve clinical benefits even with a high tumor mutational load (TMB-H or TMB ≥ 10)^7,8^. Studies have shown that specific gene mutations are associated with sensitivity of ICBs^9,10^. We hypothesized that gene mutations not only produce tumor neoantigens, but also functionally affect the clinical outcomes of immunotherapy. Thus, identifying the gene mutations associated with immunotherapy resistance is crucial for patients with NSCLC to avoid irrational drug use and for comprehensive management. In this study, we aimed to precisely identify the gene mutations associated with immunotherapy resistance and further informed mechanisms in NSCLC.

## Materials and methods

### Clinical samples and gene data acquisition

A combined cohort of 350 ICB-treated patients from Memorial Sloan Kettering Cancer Center (MSKCC) was used for testing cohort to assess the relationship between gene mutations and ICB efficacy in NSCLC^6^. In addition, we used cBioPortal to download an independent validation cohort with ICB-treated NSCLC from several other published studies in which profession-free survival (PFS) data were available^11,12^. The somatic mutation, patient prognosis information, and mRNA expression profiling data of the non-immunotherapy NSCLC cohort were downloaded from the TCGA database. The TCGA cohort was used to demonstrate that PBRM1 mutation was predictive of prognosis in patients with ICB-treated NSCLC instead of NSCLC in general and then further inform mechanisms. The clinical characteristics of the two immunotherapy cohorts were shown in Table 1.

**Table1:** Clinical characteristics of the two immunotherapy cohorts.

	MSKCC cohort	Validation cohort
No. of patients	350	256
**Age**		
21–50	34 (9.7%)	27 (10.5%)
50–60	75 (21.4)	64 (25.0%)
61–70	119 (34.0%)	86 (33.6%)
> 70	122 (34.8%)	79 (30.9%)
**Gender (%)**		
Female	180 (51.4%)	130 (50.7%)
Male	170 (48.5%)	126 (49.2%)
**Sample type**		
Primary	171 (48.8%)	NA
Metastasis	179 (51.1%)	NA
**TMB**		
High	112 (32%)	86 (33.6%)
Low	238 (68%)	170 (66.4%)
**Drug type**		
Monotherapy (PD-1/PDL-1)	329 (94.0%)	206 (80.4%)
Combination (CTLA4 and PD-1/PDL-1)	21 (6.0%)	34 (13.2%)
Unknown	0	16 (6.2%)
**OS status**		
Living	131 (37.4%)	NA
Deceased	219 (62.5%)	NA
**PFS status**		
Progressed	NA	46 (17.9%)
Not progressed	NA	210 (82%)

### Gene mutation and survival analysis

The mutational data of the MSKCC cohort were used to identify genes whose mutations could influence the prognosis of ICB using univariate and multivariate Cox regression analysis. Overall or progression-free survival of patients with or without PBRM1 mutation was then compared in the MSKCC, TCGA, and the independent ICB treatment cohort using the Kaplan–Meier method.

### Estimation of immune infiltrating pattern

Cell type identification by estimating the relative subsets of RNA transcripts (CIBERSORT) is a deconvolution algorithm based on gene expression and applies support vector regression to infer cell type proportions in data from bulk cancer samples of mixed cell types. The relative abundances of 22 immune cell types in NSCLC tissues were estimated using the CIBERSORT algorithm^13^. CIBERSORT was run with default parameters.

### Statistical analysis

The chi-square test was used to compare category variables, while the Wilcoxon test was used to examine statistical differences in numerable variables. Univariate and multivariate Cox regression analysis were performed to identify the genes whose mutations could influence the prognosis of ICB treatment. The Kaplan–Meier curve was drawn using the "Survival" package and "survminer" package in R. Statistical significance was set at *P* < 0.05. All statistical analyses were performed using the R language.

### Ethics approval

Because our data were all downloaded from public databases, there were no requirements for ethical approval. The study was conducted in accordance with the Declaration of Helsinki (as revised in 2013).

## Results

### PBRM1 mutations have independent prognostic value in patients with NSCLC with ICB treatment

**Figure 1:**
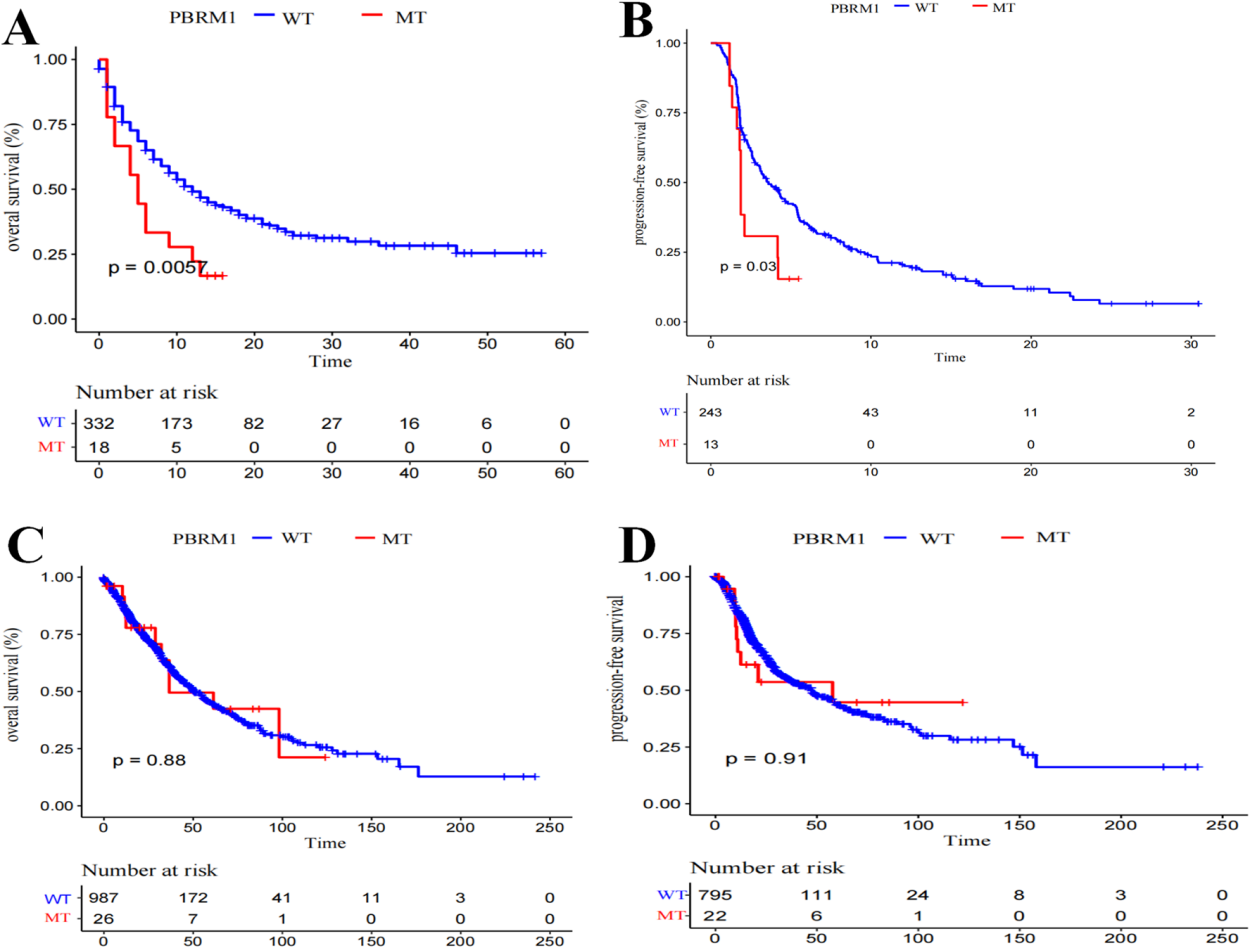
Survival analysis of NSCLC comparing patients with PBRM1 mutations and PBRM1 wild type. (**A**) Survival analysis of OS in patients with ICB-treated NSCLC. (**B**) Survival analysis of PFS in patients with ICB-treated NSCLC. (**C**) Survival analysis of OS in patients with NSCLC from the TCGA cohort. (**D**) Survival analysis of PFS in patients with NSCLC from the TCGA cohort.

A total of 350 patients with ICB-treated NSCLC from the MSKCC cohort^6^ were used to identify genes whose mutations could negatively influence immunotherapy efficacy. Gene mutations with a frequency of > 5% were included for further analysis. Univariate cox regression analysis revealed that drug type, TMB status, and eight genes were associated with patient survival (*P* < 0.05) (Table 2). Statistically significant predictors (*P* < 0.05) were introduced into the multivariate Cox regression analysis. We observed that PBRM1 mutations independently and negatively influence immunotherapy efficacy (HR 2.597; 95% CI 1.486–4.537; *P* = 0.001, Table 2). Survival analysis showed that the overall survival (OS) of NSCLC patients with PBRM1 mutation type (PBRM1-MT) was significantly shorter than that of patients with PBRM1 wild-type (PBRM1-WT) (Fig. 1A). We further compared the influence of PBRM1 mutations on PFS in patients with external NSCLC for which PFS data were available^11,12^. The data also demonstrated that PFS of NSCLC patients with PBRM1 mutations was significantly shorter than that of patients with PBRM1-WT (Fig. 1B).

**Table 2:** Prognostic value of clinical factors and the gene mutation with overall survival in NSCLC treated with ICB.

Variable	Univariate analysis			Multivariate analysis		
	HR	95% CI	*P*	HR	95% CI	*P*
Drug (Combination VS Monotherapy)	0.405	0.206–0.796	0.009	0.401	0.202–0.797	0.009
TMB (High VS Low)	0.971	0.954–0.989	0.001	0.991	0.967–1.016	0.470
EPHA3 (Mutation VS Wild)	0.580	0.354–0.953	0.031	0.717	0.427–1.204	0.209
EPHA5 (Mutation VS Wild)	0.474	0.258–0.869	0.016	0.720	0.366–1.417	0.342
EPHA7 (Mutation VS Wild)	0.337	0.139–0.819	0.016	0.564	0.226–1.408	0.220
MGA (Mutation VS Wild)	0.464	0.229–0.941	0.033	0.678	0.325–1.416	0.301
NTRK3 (Mutation VS Wild)	0.358	0.156–0.805	0.013	0.488	0.206–1.157	0.103
PBRM1 (Mutation VS Wild)	2.098	1.237–3.558	0.006	2.597	1.486–4.537	0.001
PTPRD (Mutation VS Wild)	0.624	0.398–0.979	0.040	0.863	0.514–1.447	0.576
ZFHX3 (Mutation VS Wild)	0.256	0.114–0.577	0.001	0.337	0.145–0.781	0.011

Monotherapy: anti-PD-(L)1 monotherapy; Combination: anti-PD-(L)1 monotherapy in combination with anti-CTLA-4; TMB high: TMB ≥ 10; TMB low: TMB < 10.

An important question is whether the observed negative clinical effects of PBRM1 mutations are genetically inherent. We studied a cohort of patients with NSCLC from the non-immunotherapy-treated TCGA cohort and found no differences in survival (Fig. 1C,D). These results suggested that PBRM1 mutation was predictive for patients with ICB-treated NSCLC instead of NSCLC in general.

### PBRM1 gene mutations predicted worse Immunotherapy response in NSCLC even with TMB-H

**Figure 2:**
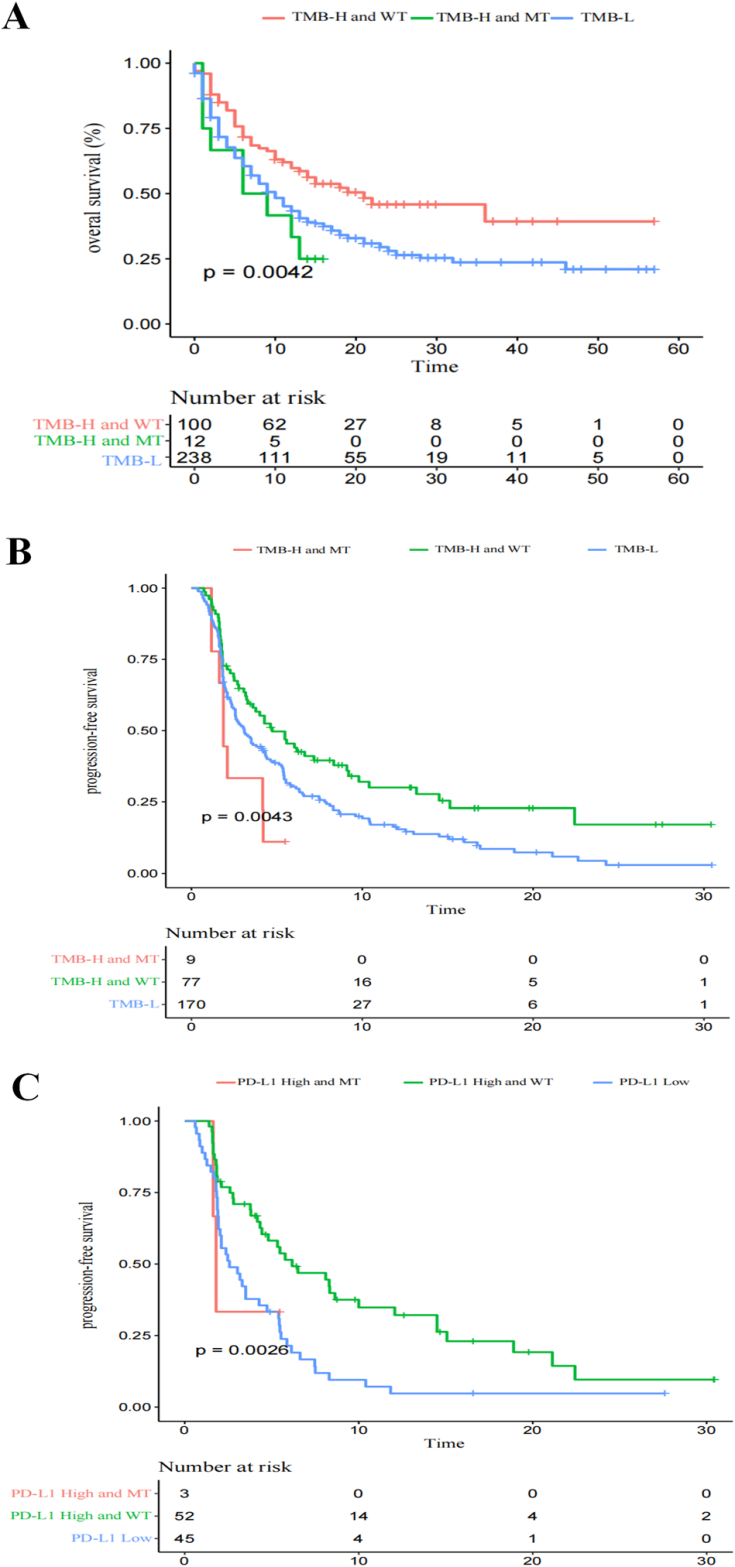
PBRM1 gene mutation predict worse immunotherapy response in NSCLC even with TMB-H. (**A**) OS levels in ICB treated NSCLC with TMB-H (≥ 10) further stratified according to groups with or without PBRM1 mutation. (**B**) PFS levels in ICB treated NSCLC with TMB-H (≥ 10) further stratified according to groups with or without PBRM1 mutation. (**C**) PFS levels of patients stratified according to their PD-L1 expression levels and PBRM1 mutations. PD-L1 low, PD-L1 expression < 1%; PD-L1 high, PD-L1 expression ≥ 1%.

A high tumor mutation load (TMB-H, or TMB ≥ 10) has been approved by the U.S. FDA as a biomarker for pembrolizumab treatment in solid tumors, including NSCLC. Therefore, we further analysed whether patients with PBRM1 mutation in the TMB-H group benefited from immunotherapy. The OS was worse in the PBRM1-MT/TMB-H group than in the PBRM1-WT/TMB-H group (*P* = 0.0408, Fig. 2A). Notably, in patients with TMB-H/PBRM1-MT, it was equal to that in patients with low TMB (TMB-L) (*P* = 0.3845, Fig. 2A). As expected, the lowest OS was observed in patients with PBRM1-MT/TMB-L (TMB-L/MT VS TMB-H/WT: *P* = 7.4e−05; TMB-L/MT VS TMB-H/MT: *P* = 0.0403; TMB-L/MT VS TMB-L/WT: *P* = 0.0078, Supplementary Fig. 1). Meanwhile, the PBRM1 mutation and PFS were similar to that of the OS data (Fig. 2B).

A sub-cohort of the external NSCLC cohort for whom the PD-L1 expression data is available was used to further study whether patients with PBRM1 mutation in the PD-L1 high group benefited from immunotherapy. Importantly, compared with PD-L1 high/WT group, patients with PBRM1 mutations had a significantly worse PFS even with high PD-L1 level (*P* = 0.0019, Fig. 2C). These results suggest that the PBRM1 mutation predicts ICB benefits independently of PD-L1 expression levels.

### Relationship between PBRM1 mutation and immune infiltrating pattern

**Figure 3:**
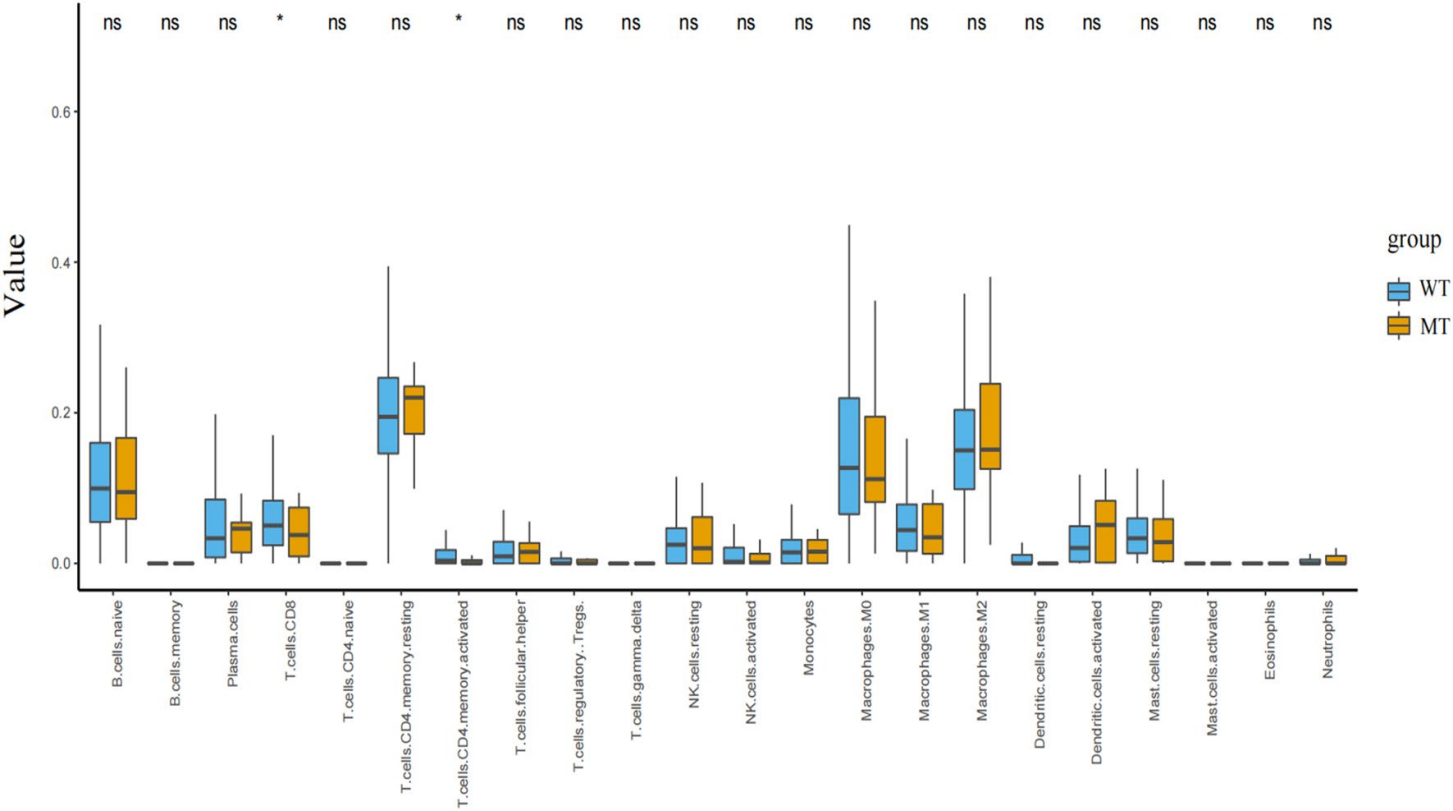
Compared the 22 types of immune cells in PBRM MT group and in WT group estimated by Cibersort algorithm.

Considering that tumor microenvironment (TME) is closely related to the efficacy of ICB in patients with NSCLC, we further evaluated immune cell infiltration in TCGA-NSCLC by CIBERSORT algorithm. We observed that the immune infiltration abundance of CD8+ T cells and activated CD4+ memory T was significantly lower in the MT group than in the WT group (Fig. 3).

### Immunogenomic features of NSCLC carrying PBRM1 mutations

**Figure 4:**
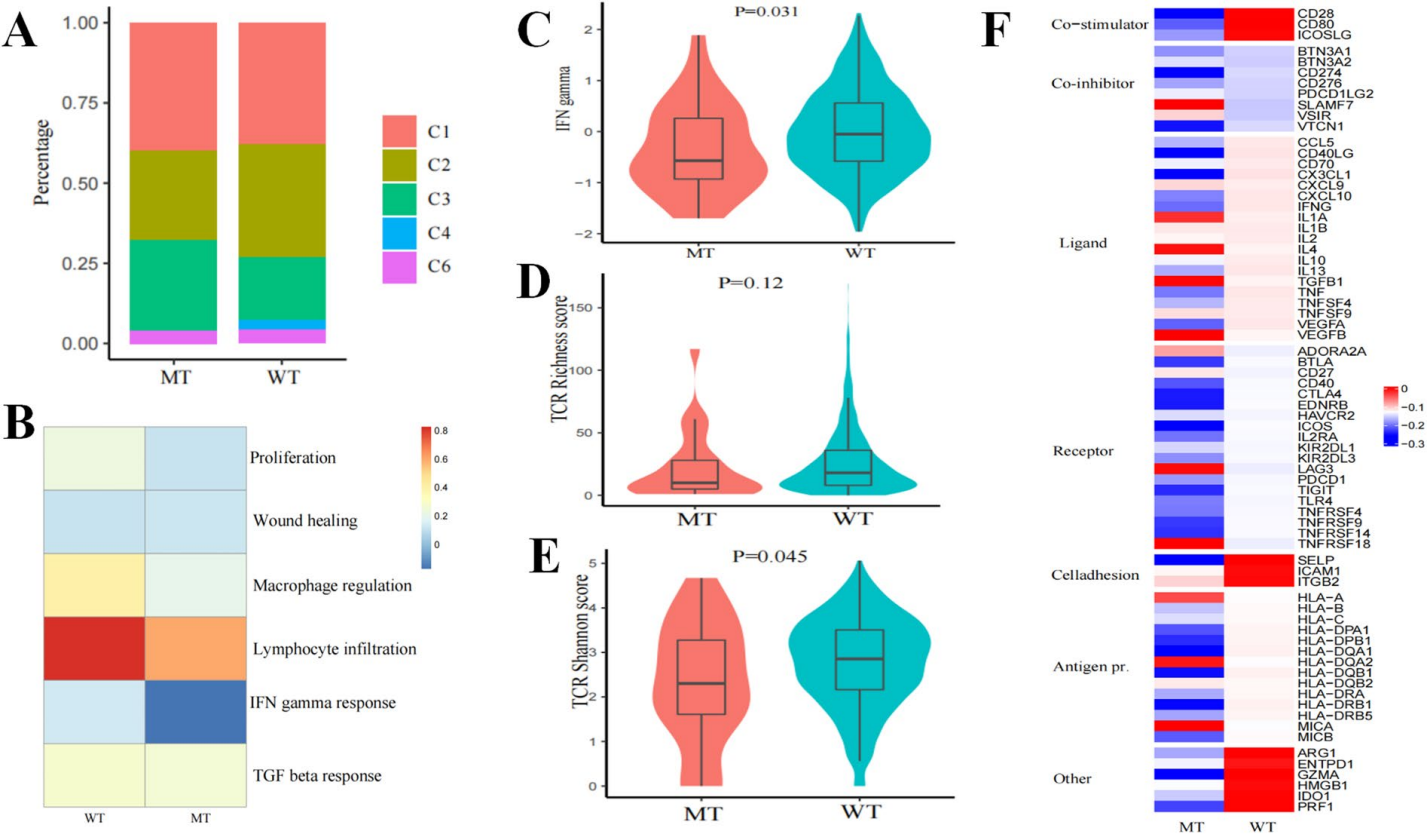
Immunogenomic frame between PBRM1 MT and WT group. (**A**) Composition of six immune subtypes. (**B**) Heatmap of immune expression signatures. (**C**) The difference of IFN-γ signature estimated by TIDE algorithm. (**D**) and (**E**) Box plots for TCR richness and TCR diversity respectively. (**F**) The expression of immune related genes in PBRM1 MT and WT group.

The immunogenomic repertoire of PBRM1 mutated NSCLC from the TCGA database was further studied. First, we compared the distribution of six immune subtypes (C1: Wound healing; C2: IFN-γ dominant; C3: Inflammatory; C4: Lymphocyte depleted; C5: Immunologically quiet; C6: TGF-β dominant)) in the two groups. Compared to the WT group, we found that C3 subtype was more widely distributed in the MT group. Conversely, the C2 subtype is less frequent in the MT group (Fig. 4A). Interestingly, C2 (IFN-γ dominant) had the highest M1 and CD8+ T cells^14^, which was associated with a better efficacy of immunotherapy.

We further analysed the differences of the immune expression signatures (proliferation, wound healing, macrophage regulation, lymphocyte infiltration, IFN-γ response, and TGF-β response) which were used to generate cancer immune taxonomy between the two groups. Macrophages/monocytes and IFN-γ response had a tendency towards higher scores in the WT group (Fig. 4B, Supplementary Fig. 2). In NSCLC, the macrophages/monocytes signatures were associated with better survival^14^. Meanwhile the IFN-γ signature was related with better efficacy of immunotherapy^15^. The TIDE algorithm^16^ further confirmed that IFN-γ signature was lower in the MT group (Fig. 4C). Moreover, TCR richness and diversity were lower in the WT group (Fig. 4D,E). Finally, the expression of co-stimulators, stimulating immune-related ligands, and antigen-presentation-related molecules were usually downregulated in MT group (Fig. 4F). Collectively, the immunogenomic frame of the WT group was consistent with its clinical outcome during ICB treatment.

### Correlation of PBRM1 mutations with tumor mutational burden and other gene mutations

WE assumed that the adverse significance in the MT group was related to TMB. Nevertheless, we noticed an inverse relationship, given that MT tumors had higher TMB than the WT group in the MSKCC and TCGA cohorts (Supplementary Fig. 3A, B). Next, we studied the relationships between PBRM1 mutation and previously confirmed genes whose mutations associated with ICB therapy efficacy: KRAS, EGFR, ALK, ZFHX3^17,18^. Our data indicated that they were evenly distributed between the two groups (Supplementary Fig. 3C).

## Discussion

ICB treatment brings dawn for advanced NSCLC^19^. Currently, TMB is the most commonly used genetic biomarker for ICB treatment in patients with NSCLC. However, some patients could not achieve clinical benefits even with a high tumor mutational load^8,20^. Screening factors affecting the efficacy of immunotherapy are keys to avoiding irrational drug use and comprehensive treatment for patients with NSCLC. Several studies have demonstrated that specific mutated genes are associated with the prognosis of immunotherapy^18,21,22^. In this study, NSCLC with PBRM1 mutation exhibited immunotherapy resistance. Survival analysis showed that the OS and PFS of patients with PBRM1 mutations were significantly shorter than those of patients with PBRM1-WT in the ICB-treated cohort. Additionally, we studied a cohort of patients with NSCLC from the non-immunotherapy-treated TCGA cohort, and found no differences in survival between the groups. These results suggest that the PBRM1 mutation was predictive for ICB-treated NSCLC instead of NSCLC in general.

A further important aspect is the unexpected short survival time of NSCLC with PBRM1 mutation despite possessing TMB-H. Patients with high TMB (≥ 10), but with PBRM1 mutation, had a significant survival disadvantage when compared with the WT/TMB-H group. Additionally, these patients could be equal to those of TMB-L patients. Although some studied have suggested that efficacy of immunotherapy increases in tumors with elevated TMB^23,24^, the association between prolonged efficacy of ICB and high TMB remains unclear^25,26^. Thus, incorporating gene mutation information into TMB may improve its capability to predict prolonged benefit from ICB in NSCLC, and similar considerations apply to PD-L1.

PBRM1 is a tumor suppressor gene in many cancer types, is involved in regulating tumor immune function, and plays an important role in tumor immunity^27–29^. Emerging studies have proved that gene mutations remodel the immune microenvironment and mediated immunosuppression^30^. In our study, we observed that the immune infiltration abundance of CD8+ T cells and activated CD4+ memory T was significantly lower in the MT group than in the WT group. It is demonstrated that CD4+ T cells are related with a better efficacy of ICB^31^. Topalian et al.^32^ reported that tumor-infiltrating lymphocytes (TILs) are associated with the responses to anti-PD1 or anti-CTLA4 therapies. Moreover, the immunogenomic features of NSCLC with PBRM1 mutation suggest an immunologically cold phenotype. These finding are consistent with the clinical outcomes of ICB treatment.

However, this study had several limitations. First, owing to data limitations, not all patients have complete clinical data records. Bias may have existed in the data analysis. Second, gene mutations were detected using targeted sequencing in the MSKCC cohort. Finally, the number of patients included in the clinical cohort was small. Prospective research of a large number of NSCLC with ICB therapy from diverse ethnics is warranted for further analysis and validation.

## Conclusions

In conclusion, our study reveals that NSCLC with PBRM1 mutations exhibited immunotherapy resistance and this result was independent of TMB. Moreover, through immune infiltrating pattern and immunogenomic feature analysis, we found that NSCLC with PBRM1 mutations might be an immunologically cold phenotype. Thus, NSCLC with PBRM1 mutation are unsuitable for immunotherapy in clinical practice. Our data helped avoid irrational immunotherapy and comprehensive treatment of NSCLC patients.

## Supplementary Information

Supplementary Figures.

## Data Availability

The data of the immunotherapy cohorts and non-immunotherapy cohort were obtained from the cBioPortal (https://www.cbioportal.org/) and TCGA (https://portal.gdc.cancer.gov/) repository, respectively. The data used to support the findings of this study are available from the corresponding author on reasonable request.
