# Supplementary material for: Non‑small cell lung cancer carrying PBRM1 mutation suggests an immunologically cold phenotype leading to immunotherapy failure even with high TMB
Source: Sci Rep. 2022 Dec 1;12:20734. doi: 10.1038/s41598-022-25050-3 (PMC9715659; doi:10.1038/s41598-022-25050-3)
Supplement: Supplementary file 1 — Supplementary Figures. [file 41598_2022_25050_MOESM1_ESM.docx]

**
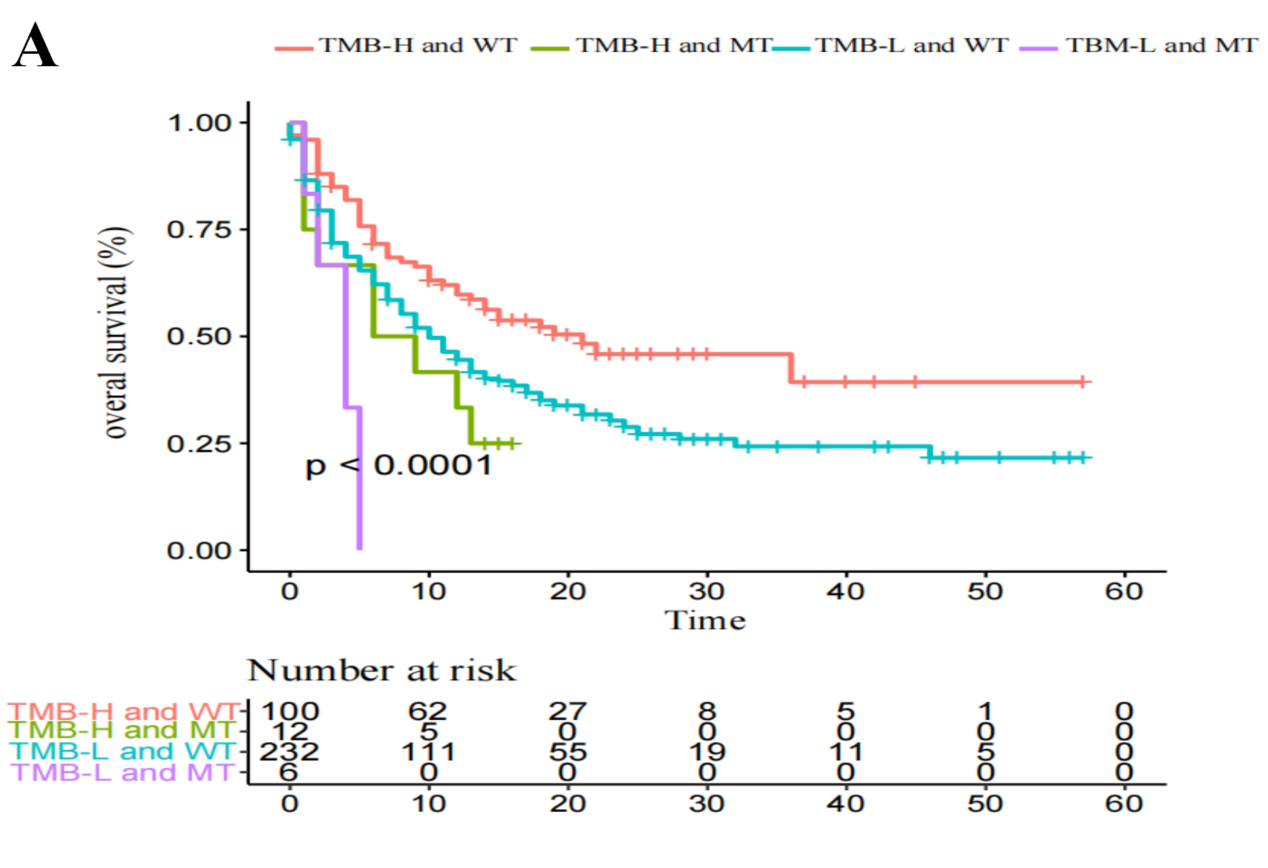
Supplementary Figure 1** OS levels in ICB treated NSCLC patients with TMB-H (≥10) and TMB-L (<10) further stratified according to groups with or without PBRM1 mutation.

**
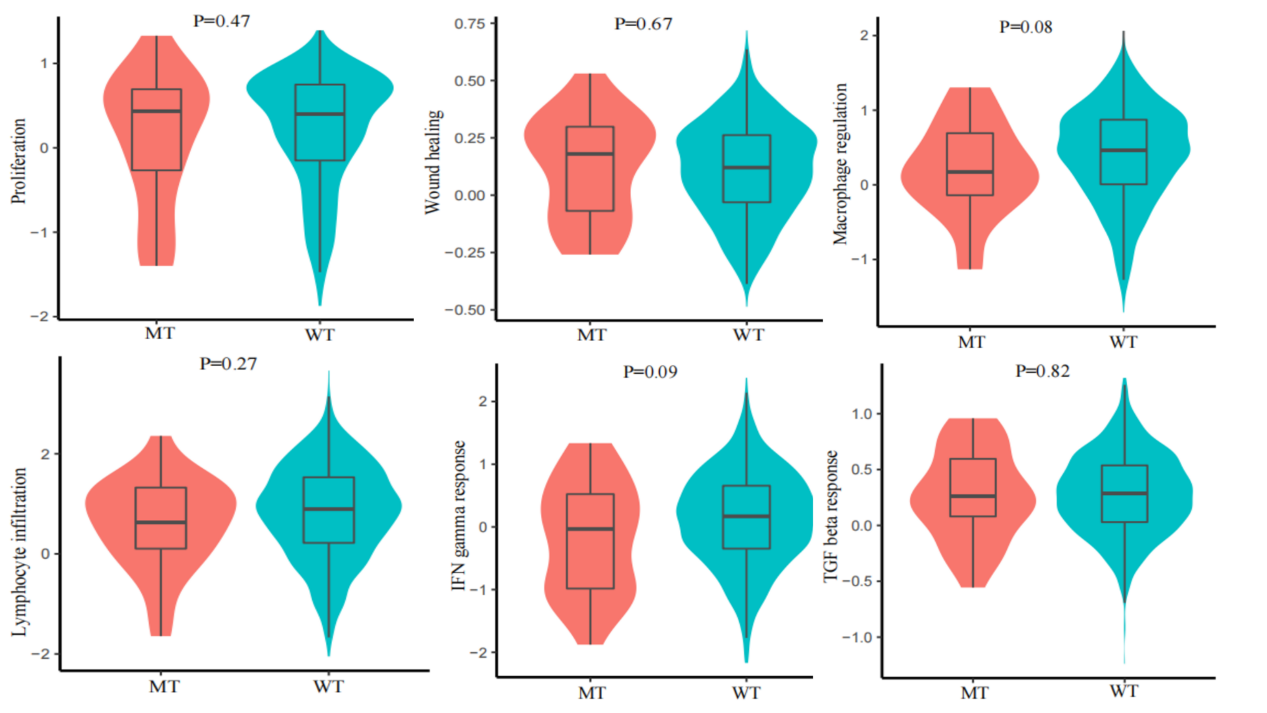
Supplementary Figure 2** The differences of the immune expression signatures between PBRM1 MT and WT group.

**
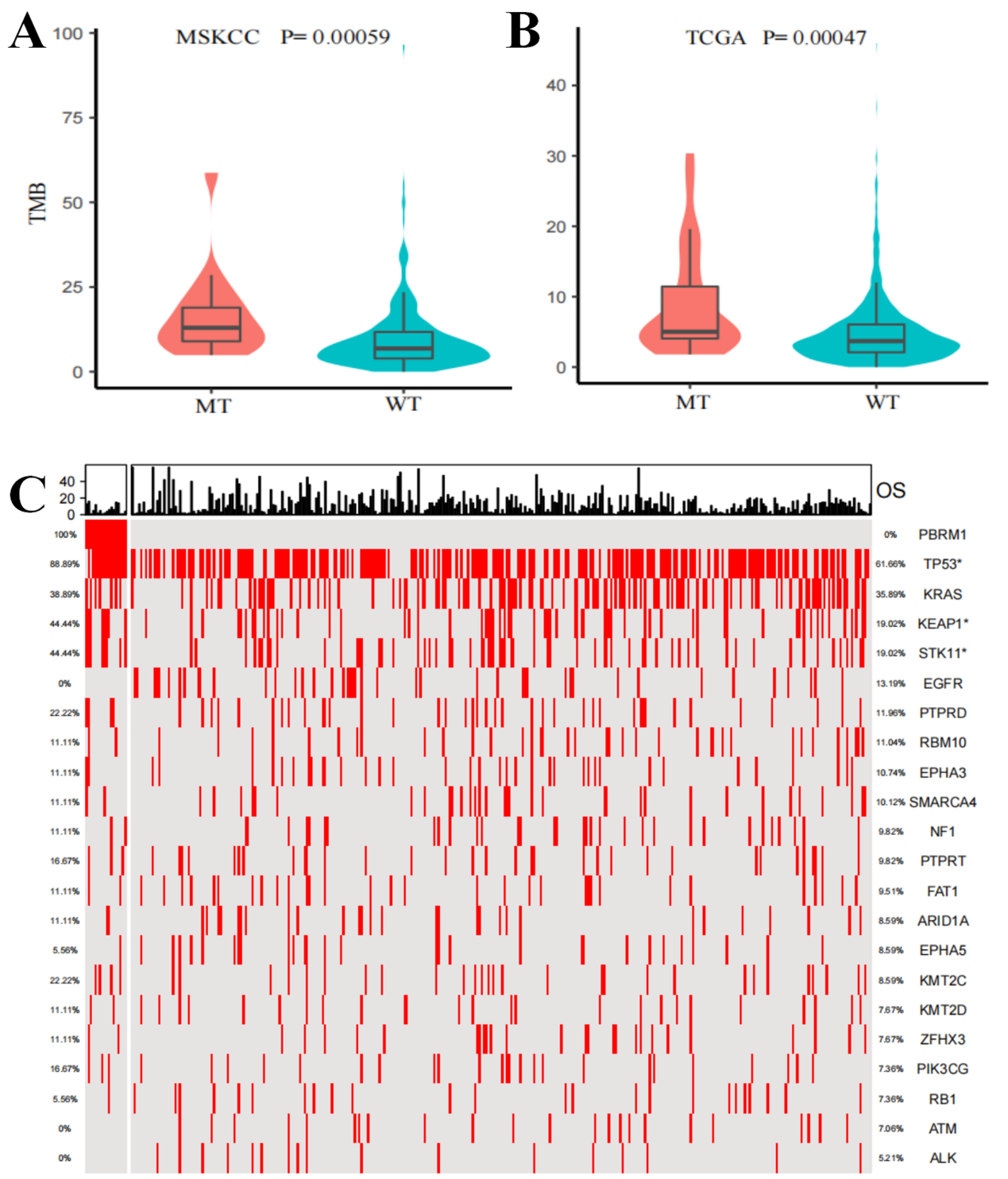
Supplementary Figure 3** Correlation of PBRM1 mutations with tumor mutational burden and other gene mutations. **A** Correlation of PBRM1 mutations with tumor mutational burden. **B** Correlation of PBRM1 mutations with other gene mutations.
